# Oregon Project Firstline: A needs assessment of healthcare personnel infection prevention knowledge and training preferences

**DOI:** 10.1017/ash.2023.348

**Published:** 2023-09-29

**Authors:** Nicholas Ida, Judith Guzman-Cottrill, Roza Tammer, Rebecca Pierce, Dat Tran

## Abstract

**Background:** Infection prevention and control (IPC) competency is critical for healthcare personnel (HCP) and patient safety. In collaboration with the CDC new national IPC training collaborative called Project Firstline, the Oregon Health Authority’s (OHA) Healthcare Associated Infection (HAI) Program established a state-level program in 2021. The goal of Oregon Project Firstline is to provide relevant, accessible, and engaging IPC training materials for our state’s HCP. We assessed the IPC learning needs of Oregon’s healthcare workforce, and to understand the preferred methods and formats of training across the various HCP roles. **Methods:** OHA’s HAI program recruited HCP by distributing electronic surveys through multiple healthcare, regulatory, and public health partners’ email listservs and HCP-targeted newsletters. Survey responses were recorded from September 23 to December 10, 2021. The HAI program assessed respondents’ IPC knowledge, online and in-person job training preferences, frequently used training devices, and trusted sources for IPC information. An individual’s understanding of an IPC topic was categorized based on their self-assessed confidence in their knowledge and ability to teach the topic to others. In total, 6,382 surveyed responses were analyzed. **Results:** The average understanding among HCP was lowest in IPC topics relating to triage and isolation of contagious patients and fit testing of respiratory protection devices. For these topics, 3,208 HCP (66.21%) and 3,657 HCP (75.48%) HCP, respectively, did not understand the topic well enough to teach others (Fig. 1). The highest number of HCP (n = 2,512, 39.36%) requested additional training in methods on how to educate others about IPC topics (ie, “train the trainer”). Surveyed respondents most frequently used personal computers for job trainings in both work and at-home settings (n = 4,603, 72.12%) and 3,437 HCP (53.85%) were open to either in-person or remote formats for job education. The CDC and OHA were the most frequented and trusted IPC sources among surveyed HCP: 4,124 HCP (64.62%) and 3,584 HCP (56.16%), respectively. **Conclusions:** IPC is a critical topic in HCP training across all healthcare facility types and employee roles. Effective educational planning includes understanding the learners’ knowledge needs and preferred methods of learning. Our learning needs assessment identified important IPC knowledge gaps and will help ensure that our training courses will be offered in effective educational formats for Oregon’s diverse HCP. Future training will include appropriate triage of potentially infectious patients, respiratory fit testing, and general IPC “train the trainer” sessions. Additionally, we will offer both in-person and remote options.

**Figure:**
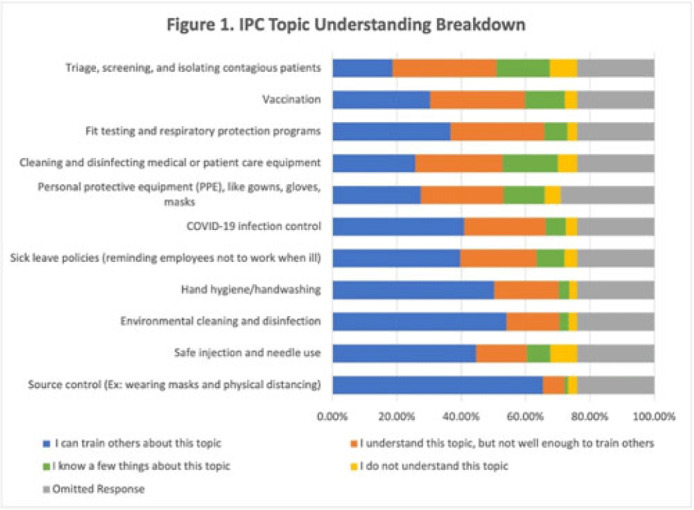

**Disclosures:** None

